# Accuracy of magnetic resonance studies in the detection of chondral and labral lesions in femoroacetabular impingement: systematic review and meta-analysis

**DOI:** 10.1186/s12891-017-1443-2

**Published:** 2017-02-16

**Authors:** A. M. Saied, C. Redant, M. El-Batouty, M. R. El-Lakkany, W. A. El-Adl, J. Anthonissen, R. Verdonk, E. A. Audenaert

**Affiliations:** 1 Department of Orthopaedic Surgery and Traumatology, Ghent University Hospital, De Pintelaan 185, B-9000 Ghent, Belgium; 2 Department of Orthopaedic Surgery, Mansoura University Hospital, Mansoura University, Mansoura, Egypt; 3 Department of Physical Medicine and Rehabilitation, Ghent University Hospital, De Pintelaan 185, B-9000 Ghent, Belgium

**Keywords:** Magnetic resonance, Hip joint, Labral lesion, Chondral lesion, Cartilage defect, Sensitivity, Specificity, Femoroacetabular impingement

## Abstract

**Background:**

Several types of Magnetic resonance imaging (MRI) are commonly used in imaging of femoroacetabular impingement (FAI), however till now there are no clear protocols and recommendations for each type. The aim of this meta-analysis is to detect the accuracy of conventional magnetic resonance imaging (cMRI), direct magnetic resonance arthrography (dMRA) and indirect magnetic resonance arthrography (iMRA) in the diagnosis of chondral and labral lesions in femoroacetabular impingement (FAI).

**Methods:**

A literature search was finalized on the 17th of May 2016 to collect all studies identifying the accuracy of cMRI, dMRA and iMRA in diagnosing chondral and labral lesions associated with FAI using surgical results (arthroscopic or open) as a reference test. Pooled sensitivity and specificity with 95% confidence intervals using a random-effects meta-analysis for MRI, dMRA and iMRA were calculated also area under receiver operating characteristic (ROC) curve (AUC) was retrieved whenever possible where AUC is equivocal to diagnostic accuracy.

**Results:**

The search yielded 192 publications which were reviewed according inclusion and exclusion criteria then 21 studies fulfilled the eligibility criteria for the qualitative analysis with a total number of 828 cases, lastly 12 studies were included in the quantitative meta-analysis.

Meta-analysis showed that as regard labral lesions the pooled sensitivity, specificity and AUC for cMRI were 0.864, 0.833 and 0.88 and for dMRA were 0.91, 0.58 and 0.92. While in chondral lesions the pooled sensitivity, specificity and AUC for cMRI were 0.76, 0.72 and 0.75 and for dMRA were 0.75, 0.79 and 0.83, while for iMRA were sensitivity of 0.722 and specificity of 0.917.

**Conclusions:**

The present meta-analysis showed that the diagnostic test accuracy was superior for dMRA when compared with cMRI for detection of labral and chondral lesions.

The diagnostic test accuracy was superior for labral lesions when compared with chondral lesions in both cMRI and dMRA. Promising results are obtained concerning iMRA but further studies still needed to fully assess its diagnostic accuracy.

**Electronic supplementary material:**

The online version of this article (doi:10.1186/s12891-017-1443-2) contains supplementary material, which is available to authorized users.

## Background

Femoroacetabular impingement (FAI) becomes a well-established syndrome with characteristic clinical and radiological findings [1]. The condition shows pathological repetitive impingement of the surrounding soft tissue structures mostly in the labrum and the adjacent cartilage leading to their damage and appearance of pain. It has been associated both with specific morphotypes as well as with extreme/repetitive motion (e.g. kickboxing and soccer) [2–4].

Two different types of FAI morphology have been described. Firstly, Cam type morphology which is characterized by a non-spherical portion of the femoral head (including the pistol-grip deformity, decreased head-neck offset, increased alpha angle, overgrowth of the femoral head epiphysis and subclinical slipped epiphysis). The second is pincer type morphology which is characterized by anterior over coverage of the acetabulum (including coxa profunda, acetabular retroversion, and lateral rim lesions). Most symptomatic hips, however, have been reported as mixed morphology and both femoral (cam) and acetabular (pincer) factors are present [1, 2, 5].

Both morphotypes are highly prevalent in asymptomatic populations reaching 30% in some studies. This indicates that the presence of this morphology is not always a pathological finding that needs interference [3, 6–10]. The precise diagnosis of FAI may therefore be difficult because both clinical examinations and plain radiographs have limited reliability in identification of labral and chondral damage [5].

Magnetic resonance imaging (MRI) in general has superior soft tissue contrast and reliability in assessing of acetabular labrum and articular cartilage of the hip. In this respect different scanning protocols have been developed for the evaluation of FAI, including conventional magnetic resonance imaging (cMRI), direct magnetic resonance arthrography (dMRA) and indirect magnetic resonance arthrography (iMRA).

To date, a gold standard has not been well established, as several studies comparing the accuracy of these different protocols obtained variable outcomes [11–14]. Moreover, there is a debate about whether introduction of contrast material increases the accuracy of cMRI or not. Introduction of contrast material may be done directly by intra-articular injection into the joint as in dMRA or indirectly by intravenous injection as in iMRA [15–19].

More recently, biochemical imaging analysis of chondral surface has shown good results for diagnosis of early abnormalities. In the delayed Gadolinium Enhanced Magnetic Resonance Imaging of Cartilage (dGEMRIC) technique which is a common protocol of iMRA, introduction of an intravenous dose of gadolinium is done, followed by a short period of exercise then subsequent imaging. Early images can be used to determine cartilage morphology and delayed images can be obtained to assess biochemical structure [20].

There are some potential advantages for iMRA over dMRA, it is simple and less invasive procedure than dMRA and may be more accepted by patients, also iMRA can be easily arranged and performed at any imaging facility [21].

In 2011 Smith et al. [19] did a meta-analysis about the accuracy of cMRI and dMRA in diagnosing acetabular labral tears, but they included all pathologies of labral tears with no specificity to FAI and they didn’t include iMRA as a valuable method in diagnosing labral tears as in this review.

In 2011 Smith et al. [18] did another meta-analysis about the accuracy of cMRI, MRA and computer tomography in diagnosing chondral lesions of the hip, but they also included all pathologies of chondral lesions with no specificity to FAI and they didn’t include iMRA as a valuable method in diagnosing chondral lesions as in this review. There was some heterogeneity in their review meta-analysis by pooling results from studies using different magnetic resonance (MR) strength fields, we also noticed that they included a study in their meta-analysis about knee not hip [22].

Till now there are no clear protocol and recommendations for MRI in diagnosing FAI and this more evident as in regard to the associated chondral lesions. About 6 years ago Smith et al. [18, 19] did their search for review meta-analysis; we reviewed the current evidence about the accuracy of conventional MRI, dMRA and iMRA in the detection of chondral and labral lesions in FAI.

## Methods

We followed the Preferred Reporting Items for Systematic Reviews and Meta-Analyses (PRISMA) [23] statement.

A thorough search of the literature was conducted and was completed on the17th of May 2016.

The primary database used was the Medical Literature Analysis and Retrieval System Online (Medline) (via PubMed) and additional data base used was Web of Science from their inception to the search date. The search was supplemented by hand-searching of databases and references of relevant articles and reviews. The search strategy was a combination of Medical Subject Headings (MeSH) terms and free text words which is represented in Table 1.

**Table 1:** Search strategy developed for PubMed and modified appropriately for other databases

FAI	#1 “femoracetabular impingement” [MeSH Terms] #2 “femoracetabular” [All Fields] AND “impingement” [All Fields]) OR “femoracetabular impingement” [All Fields] OR “femoroacetabular” [All Fields] AND “impingement” [All Fields]) OR “femoroacetabular impingement” [All Fields]
Hip joint	#3 “hip joint”[MeSH Terms] OR (“hip” [All Fields] AND “joint” [All Fields]) OR “hip joint” [All Fields] #4 chondral [All Fields] OR “cartilage” [MeSH Terms] OR “cartilage” [All Fields] #5 labral [All Fields] OR acetabular [All Fields] AND labrum [All Fields]
MRI	#6 “magnetic resonance imaging” [MeSH Terms] OR (“magnetic” [All Fields] AND “resonance” [All Fields] AND “imaging” [All Fields]) OR “magnetic resonance imaging” [All Fields] #7 (“magnetic resonance spectroscopy” [MeSH Terms] OR (“magnetic” [All Fields] AND “resonance” [All Fields] AND “spectroscopy” [All Fields]) OR “magnetic resonance spectroscopy” [All Fields] OR (“magnetic” [All Fields] AND “resonance” [All Fields]) OR “magnetic resonance” [All Fields]) AND (“arthrography” [MeSH Terms] OR “arthrography” [All Fields])
Accuracy of MRI	#8 (“sensitivity and specificity” [MeSH Terms] OR (“sensitivity” [All Fields] AND “specificity” [All Fields]) OR “sensitivity and specificity” [All Fields] OR (“sensitivity” [All Fields] AND “specificity” [All Fields]) OR “sensitivity specificity” [All Fields]) AND accuracy [All Fields] #9 (true [All Fields] AND positive [All Fields]) OR (true [All Fields] AND negative [All Fields]) OR (false [All Fields] AND positive [All Fields]) OR (false [All Fields] AND negative [All Fields])
Search strategy	((#1 AND #2) AND (#3 OR #4 OR #5)) AND ((#6 OR #7) OR (#8 OR #9))
Last search	17th of May 2016

### Eligibility criteria

All studies reporting the diagnostic test accuracy (sensitivity/specificity) of cMRI, dMRA and iMRA for the assessment of chondral and labral lesions in FAI with surgical comparison (open or arthroscopic) as the reference test, were included.

### Inclusion criteria

1. Publications must be reported in International Peer-reviewed Journals with English abstract.
2. All studies handling data on chondral and labral lesions in FAI were included even if the paper investigated and presented a wider range of other hip joint pathology.
3. All studies must include cMRI or dMRA or iMRA as diagnostic tests with surgical comparison as the reference test.
4. Studies in which sensitivity and specificity are mentioned.

### Exclusion criteria

1. Studies assessing cadaver or animal specimens.
2. Articles describing other studies.
3. Studies assessing revision surgery.
4. Case reports.

### Study identification

All search data were collected and initial screening of the abstracts was performed by one reviewer (Saied AM) based on inclusion and exclusion criteria. Full-text documents were obtained for all studies meeting the criteria above then further analysis was done by two reviewers (Saied AM., Audenaert EA), Audenaert EA checked the data collected by Saied AM then final agreement on the final data between the 2 investigators was obtained.

The data extracted included Country of study, sample size, mean age, type of magnetic resonance procedure, type of lesion analyzed (Acetabular chondral delamination, Combined chondral lesions, Femoral head chondral lesions, Acetabular chondral lesions and labral lesions), sensitivity and specificity for each type of lesion.

Also the frequency of true-positives (TP), true-negatives (TN), false-positives (FP) and false-negatives (FN) for the MRI studies to the reference test were collected to perform statistical meta-analysis. If insufficient, attempts were made to estimate the values and if this was not possible the study was excluded from the meta-analysis.

Quality Assessment of Diagnostic Accuracy Studies 2 (QUADAS-2) criteria was used to assess all studies for their methodological quality [24]. QUADAS-2 tool shows improved criteria, it distinguishes between bias and applicability and identifies 4 key domains supported by signaling questions to aid assessing risk of bias and concerns about applicability as “high” and “low”.

For risk of bias signaling questions are answered as “yes,” “no,” or “unclear” and are handled such that “yes” indicates low risk of bias. Risk of bias is determined as “low,” “high,” or “unclear”. If the answers to all signaling questions for a domain are “yes,” then risk of bias can be determined low. If any signaling question is answered “no,” potential for bias occurs. The “unclear” category should be used only when insufficient data are reported to permit a judgment.

Applicability concerns shows if the study matches the review question or not, so systematic review question in terms of patients, index tests, and reference standard must be reported. Concerns about applicability are rated as “low,” “high,” or “unclear”. The “unclear” result should be used only when insufficient data are reported [24].

Meta-analysis was done by assessing the pooled sensitivity and specificity with 95% confidence intervals using random effect. Only studies with similar types of MRI, strength of magnetic field machines and lesion types were included. Also area under receiver operating characteristic (ROC) curve (AUC) [25] was retrieved whenever possible where AUC represented the diagnostic accuracy. AUC values are graded as the following:

- 0.9 – 1.0 excellent
- 0.8 – 0.9 very good
- 0.7 – 0.8 good
- 0.6 – 0.7 sufficient
- 0.5 – 0.6 bad
- <0.5 test not useful

All analysis was done on SPSS version 18.0 (SPSS Inc, Chicago, Illinois) and Meta-Disc (Unit of Clinical Biostatistics, Ramo´ny Cajal Hospital, Madrid, Spain) [26] (Additional files 1, 2, 3 and 4).

## Results

**Fig. 1:**
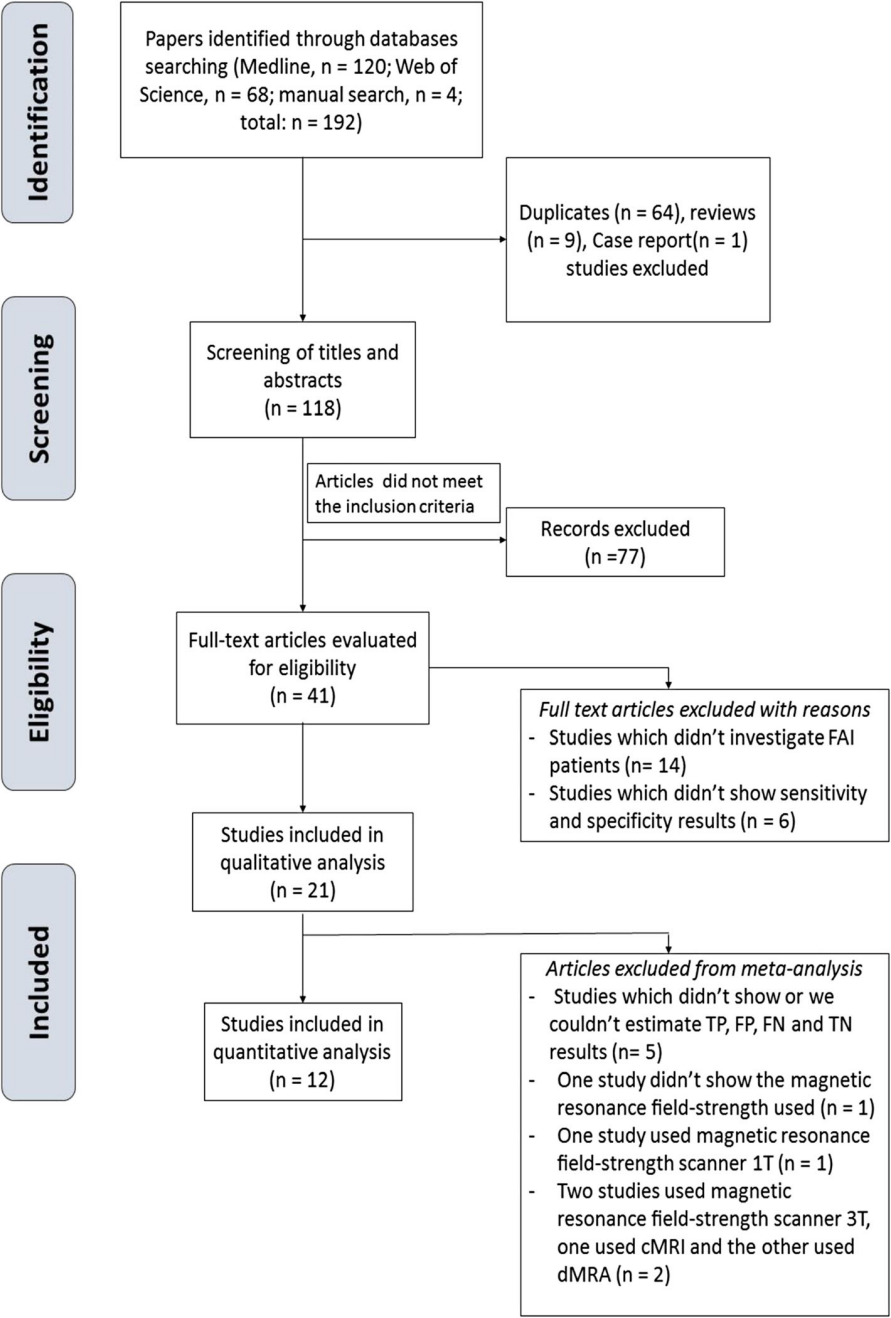
PRISMA flow-chart

The results of literature search strategy are illustrated in the PRISMA flowchart (Fig. 1). A total number of 192 papers were collected from which 21 studies met the eligibility, inclusion and exclusion criteria and were included in the qualitative analysis using QUADAS-2 criteria.

Only 12 studies were included in the quantitative meta-analysis, five studies were excluded because there was no available data as regard TP, TN, FP, FN [21, 27–30] (Table 3). To decrease heterogeneity of data, another 4 studies were excluded because it wasn’t suitable to pool their results with another studies, one study didn’t show the used MR field-strength [17], another study used MR field-strength scanner 1 T [15] and two studies used MR field-strength scanner 3 T (one used cMRI [14] and the other used dMRA [31]) (Fig. 1).

### Qualitative analysis

The results of the QUADAS-2 showed that all studies had low risk for applicability concerns. There was some variation in the results for the risk of bias specially for the description of time between MRI and surgery. Nine studies didn’t describe the time between MRI and surgery while it was mentioned in 12 studies; in 5 studies the time interval exceeded 3 months in some cases while the other 7 studies the time interval was below 3 months. Most studies showed that the surgical procedures were done with the knowledge of the radiological findings and sometimes clinical data were available to radiologists when they reviewed the images.

Most of studies showed high risk of bias in identification of cohort recruitment, all studies showed that the patients received both the reference (surgery) and index tests (MRI) and that the surgery was independent of the MRI. All studies showed that MRI interpretation was done without the knowledge of the surgical findings (Table 2).

**Table 2:**
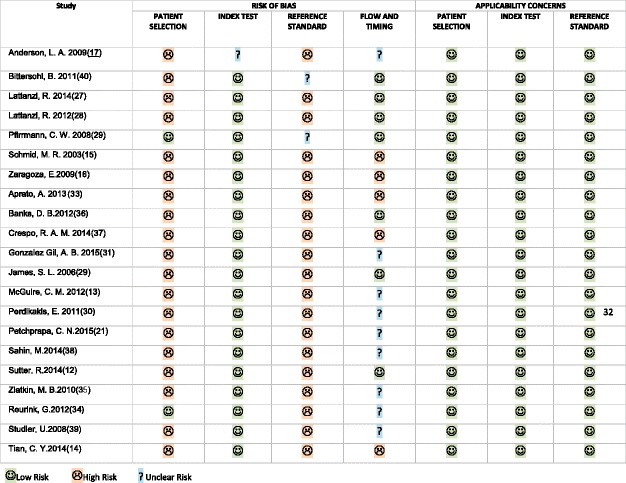
QUADAS-2 tool results

### Study demographics

A total number of 828 cases were determined. Mean age of the study cohorts was 34.4 years, mentioned in 19 studies. This ranged from a mean value of 19 to 43 years. The time from radiological assessment to surgical comparison was documented in 12 studies. This ranged from within 3 days to within 6 months [12, 14–16, 21, 27, 28, 32–34].

The diagnostic test accuracy of cMRI was evaluated in 5 studies. Of these, one study used a 3-Tesla (T) field strength magnet [14], while the other 4 studies evaluated MRI using a 1.5-T magnet [12, 13, 29, 35]. Fifteen studies evaluated the diagnostic test accuracy of dMRA, this was done with a 1.0-T magnet in 1 study [15], 1.5-T magnet in 11 studies [12, 13, 16, 30, 32–34, 36–39] and 3 T magnet in 2 studies [14, 31] and the field strength of the MRI machine was not stated in one study [17]. Five studies assessed the diagnostic test accuracy of iMRA one with 3 T magnet [21] and the others with 1.5 T magnet [27, 28, 35, 40]. The radiological images were reviewed by musculoskeletal radiologists in all studies.

All sensitivities and specificities of cMRI, dMRA and iMRA were retrieved and organized in relation to study ID and type of lesions analyzed (Acetabular chondral delamination, combined chondral lesions, Femoral head chondral lesions, Acetabular chondral lesions and labral lesions) (Table 3).

**Table 3:** Study demographics

Study	Country	Number of cases	Mean age (years)	MR procedure	Lesion analyzed	Sensitivity (percent)	Specificity (percent)	TP (n)	FP (n)	FN (n)	TN (n)
Anderson, L. A. 2009 [17]	USA	27	N/S	*dMRA*	*Acetabular chondral delamination*	22	100	2	2	7	18
Bittersohl, B. 2011 [40]	Switzerland	16	31	*iMRA 1.5 T (morphologic)*	*Combined chondral lesions*	57	88	4	1	3	8
				*iMRA 1.5 T (dGEMRIC)*	*Combined chondral lesions*	75	33	5	6	2	3
Lattanzi, R. 2014 [27]	USA	20	N/S	iMRA 3 T*(dGEMRIC)*	*Combined chondral lesions*	52	67	N/S	N/S	N/S	N/S
Lattanzi, R. 2012 [28]	USA	10	19.9	*iMRA 1.5 T (morphologic)*	*Combined chondral lesions*	47	79	N/S	N/S	N/S	N/S
				*iMRA 1.5 T (dGEMRIC)*	*Combined chondral lesions*	71	36	N/S	N/S	N/S	N/S
Pfirrmann, C. W. 2008 [32]	Switzerland	44	30.7 (16–49)	*dMRA 1.5 T*	*Acetabular chondral delamination*	74	90	17	2	6	19
Schmid, M. R. 2003 [15]	Switzerland	42	37	dMRA 1.0 T	*Combined chondral lesions*	79	77	15	5	4	18
					*Femoral head chondral lesions*	60	88	N/S	N/S	N/S	N/S
Zaragoza, E. 2009 [16]	Canada, USA	48	38.8	dMRA 1.5 T	*Acetabular chondral delamination*	79	84	28	3	1	16
Aprato, A. 2013 [33]	Italy	41	23-25	dMRA 1.5 T	*Acetabular chondral lesions*	69	88	11	5	3	22
					*Femoral head chondral lesions*	46	81	6	8	5	22
					*Labral lesions*	91	86	31	3	1	6
Banks, D. B. 2012 [36]	United Kingdom	69	N/S	dMRA 1.5 T	*Combined chondral lesions*	17	100	6	0	29	34
					Labral lesions	81	51	13	26	3	27
Crespo Rodriguez, A. M. 2014 [37]	Spain	51	43 ± 9	dMRA 1.5 T	*Combined chondral lesions*	92	54	37	5	3	6
					*Labral lesions*	95	100	35	0	2	14
Gonzalez Gil, A. B. 2015 [31]	Spain	36	39	dMRA – 3 T	*Acetabular chondral lesions*	78.78	81.81	11	4	3	18
					*Femoral head chondral lesions*	71.43	72.72	10	6	4	16
					*Labral lesions*	86.95	76.92	20	3	3	10
James, S. L. 2006 [29]	United Kingdom, Australia	46	32.3	cMRI – 1.5 T	*Acetabular chondral lesions*	94	100	N/S	N/S	N/S	N/S
					*Femoral head chondral lesions*	100	94	N/S	N/S	N/S	N/S
					*Labral lesions*	100	100	N/S	N/S	N/S	N/S
McGuire, C. M. 2012 [13]	Ireland	61	32	dMRA – 1.5 T (31 cases)	*Acetabular chondral lesions*	86	50	25	1	4	1
					*Femoral head chondral lesions*	85	44	12	8	3	8
					*Labral lesions*	96	33	25	4	1	1
				cMRI – 1.5 T (30 cases)	*Acetabular chondral lesions*	78	33	21	2	6	1
					*Femoral head chondral lesions*	69	43	10	9	5	6
					*Labral lesions*	86	100	25	0	4	1
Perdikakis, E. 2011 [30]	Greece	14	43	dMRA 1.5 T	*Combined chondral lesions*	63	33	N/S	N/S	N/S	N/S
					*Labral lesions*	100	50	N/S	N/S	N/S	N/S
Petchprapa, C. N. 2015 [21]	USA	41	33-35	iMRA 3 T	*Acetabular chondral lesions*	69	89	N/S	N/S	N/S	N/S
					*Femoral head chondral lesions*	69	95	N/S	N/S	N/S	N/S
					*Labral lesions*	89	99	N/S	N/S	N/S	N/S
Sahin, M. 2014 [38]	Turkey	14	35	dMRA – 1.5 T	*Acetabular chondral lesions*	89	40	8	3	1	2
					*Femoral head chondral lesions*	100	90	4	1	0	9
					*Labral lesions*	100	50	10	2	0	2
Sutter, R. 2014 [12]	Switzerland	28	31.8	dMRA – 1.5 T	*Acetabular chondral lesions*	91	25	22	3	2	1
					*Femoral head chondral lesions*	50	90	3	2	3	20
					*Labral lesions*	89	50	23	1	3	1
				cMRI – 1.5 T	*Acetabular chondral lesions*	83	50	20	2	4	2
					*Femoral head chondral lesions*	50	100	3	0	3	22
					*Labral lesions*	89	50	23	1	3	1
Zlatkin, M. B. 2010 [35]	USA	14	39.0	iMRA 1.5 T	*Combined chondral lesions*	81	100	9	0	2	3
					*Labral lesions*	100	100	13	1	0	0
				cMRI 1.5 T	*Combined chondral lesions*	81	100	9	0	2	3
					*Labral lesions*	84	100	11	0	2	1
Reurink, G. 2012 [34]	Netherlands	95	41.3	dMRA 1.5 T	*Labral lesions*	86	75	78	1	13	3
Studler, U. 2008 [39]	Switzerland	57	35.0	dMRA 1.5 T	*Labral lesions*	97	53	43	6	1	7
Tian, C. Y. 2014 [14]	China	90	35.1	dMRA 3 T	*Labral lesions*	95	84	N/S	N/S	N/S	N/S
				cMRI 3 T	*Labral lesions*	66	77	39	7	20	24

*cMRI* conventional magnetic resonance imaging, *dMRA* direct magnetic resonance arthrography, *iMRA* indirect magnetic resonance arthrography, *N/S* not stated, *TP* T tesla, true-positives, *TN* true-negatives, *FP* false-positives, *FN* false-negatives, *dGEMRIC* Delayed Gadolinium Enhanced Magnetic Resonance Imaging of Cartilage

### Quantitative meta-analysis

#### Conventional magnetic resonance imaging

**Fig. 2:**
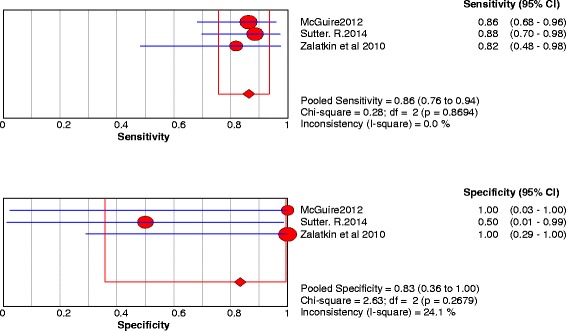
Forest plot of sensitivity and specificity for the diagnostic accuracy of conventional magnetic resonance imaging for detecting acetabular labral lesions

**Fig. 3:**
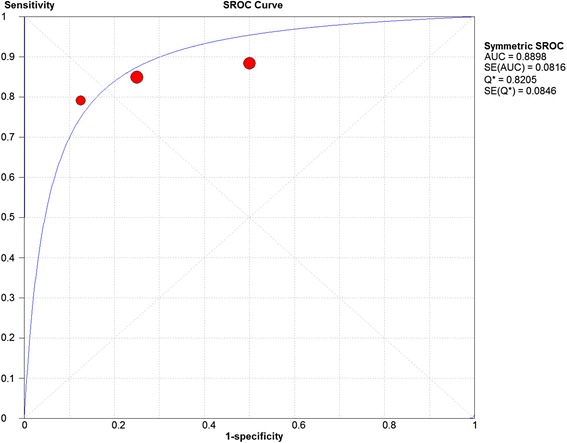
A SROC diagram assessing the sensitivity and specificity for the diagnostic accuracy of conventional magnetic resonance imaging for detecting acetabular labral lesions
B) Chondral lesions
  Three studies were included and 5 data sets were retrieved. The individual sensitivity and specificity results are presented in Fig. 4. The summary ROC diagram (Fig. 5) showed AUC of 0.75. The pooled analysis indicated Sensitivity of 0.76 (95% CI: 0.65–0.85), specificity of 0.72 (95% CI: 0.57–0.84) (Table 4).

A) Labral lesions
  Three studies were included. The sensitivity and specificity results for each study are illustrated in Fig. 2. The results showed some variation between studies and this was reflected in the summary ROC diagram (Fig. 3). The pooled analysis indicated Sensitivity of 0.864 (95% CI: 0.757 – 0.936), specificity of 0.833 (95% CI: 0.359– 0.996) and AUC of 0.88 (Table 4).

**Fig. 4:**
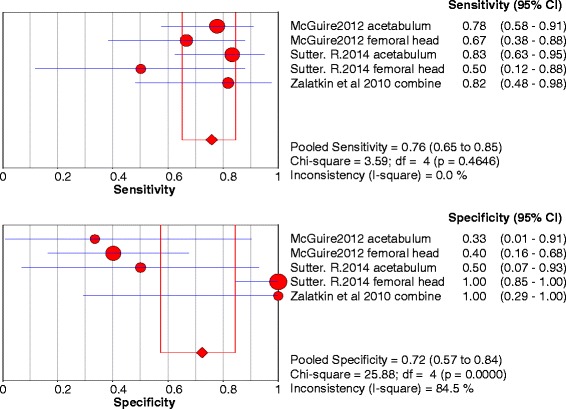
Forest plot of sensitivity and specificity for the diagnostic accuracy of conventional magnetic resonance imaging for detecting chondral lesions

**Fig. 5:**
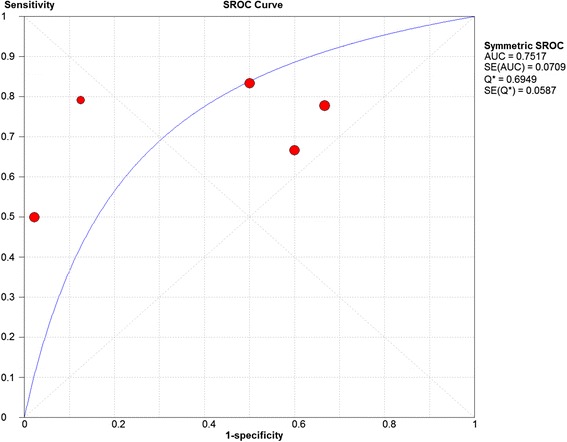
A SROC diagram assessing the sensitivity and specificity for the diagnostic accuracy of conventional magnetic resonance imaging for detecting chondral lesions

#### Direct magnetic resonance arthrography

**Fig. 6:**
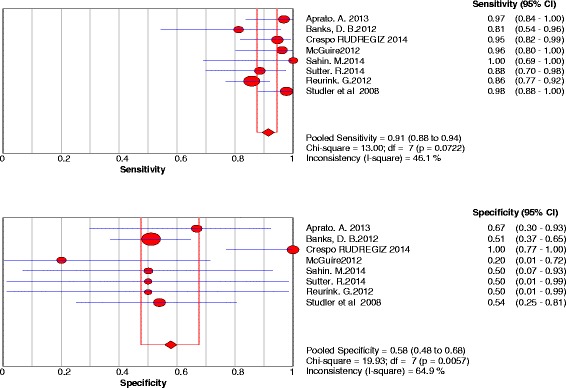
Forest plot of sensitivity and specificity for the diagnostic accuracy of direct magnetic resonance arthrography for detecting acetabular labral lesions

**Fig. 7:**
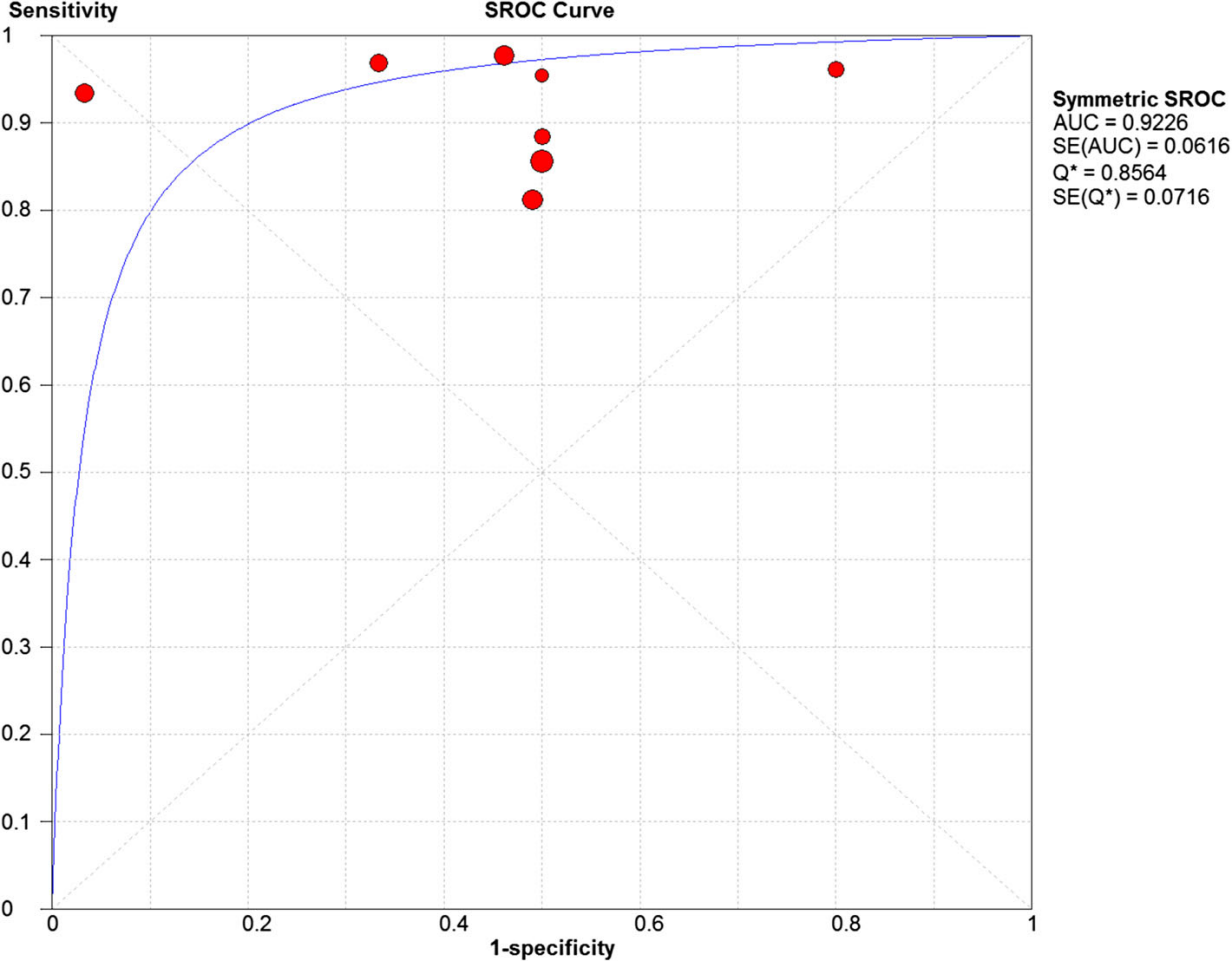
A SROC diagram assessing the sensitivity and specificity for the diagnostic accuracy of direct magnetic resonance arthrography for detecting acetabular labral lesions
B) Chondral lesions
  Eight studies were included and 12 data set were retrieved. The individual sensitivity and specificity results are presented in Fig. 8. The summary ROC diagram (Fig. 9) showed AUC of 0.83. The pooled analysis indicated sensitivity of 0.75 (95% CI: 0.69 – 0.8), specificity of 0.79 (95% CI: 0.73 – 0.85) (Table 4).

A) Labral lesions
  Eight studies were included. The sensitivity and specificity results for each MRI study are illustrated in Fig. 6. The summary ROC diagram (Fig. 7) showed AUC of 0.92. The pooled analysis indicated Sensitivity of 0.91 (95% CI: 0.88 – 0.94), specificity of 0.58(95% CI: 0.48–0.68) (Table 4).

**Fig. 8:**
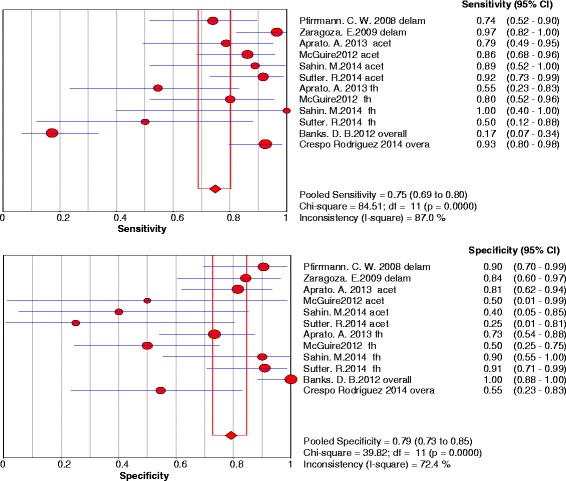
Forest plot of sensitivity and specificity for the diagnostic accuracy of direct magnetic resonance arthrography for detecting chondral lesions

**Fig. 9:**
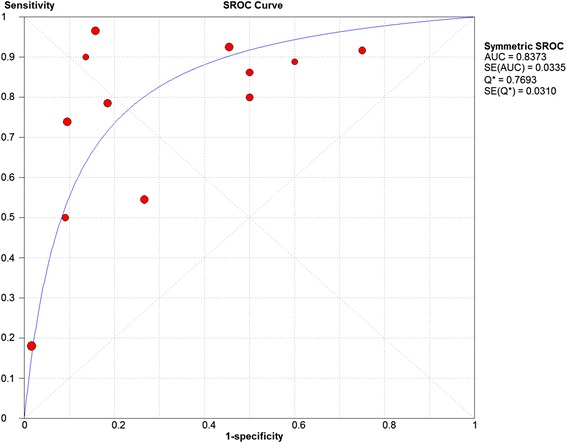
A SROC diagram assessing the sensitivity and specificity for the diagnostic accuracy of direct magnetic resonance arthrography for detecting chondral lesions

#### Indirect magnetic resonance arthrography

##### Chondral lesions

**Fig. 10:**
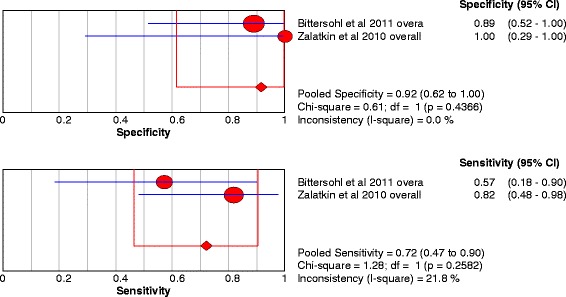
Forest plot of sensitivity and specificity for the diagnostic accuracy of indirect magnetic resonance arthrography for detecting chondral lesions

Two studies were included and 2 data sets were retrieved. The individual sensitivity and specificity results are presented in Fig. 10. The pooled analysis indicated Sensitivity of 0.722 (95% CI: 0.465 – 0.903), specificity of 0.917 (95%CI: 0.615 – 0.998) (Table 4).

## Discussion

The results of the QUADAS-2 assessment showed that about 45% of studies didn’t mention the duration interval between MRI and surgery, about 23% of studies mentioned this duration with limit reaching 6 months. This could increase the possibility that the patient chondral or labral condition change between the index and reference tests. However, 35% of studies mentioned this duration with limit reaching 3 months which considered accepted duration (Table 2).

The results for patient selection showed low risk of applicability concerns in all studies, this could be explained by specifying FAI patients for the eligibility criteria in this review. However, it showed high risk of bias in most studies where only 2 studies [32, 34] showed that a consecutive or random sample of patients were enrolled. However, all studies avoided case control design and avoided inappropriate exclusions (Table 2).

The results of the QUADAS-2 assessment also showed that all studies showed low risk of applicability concerns as in regard to the reference standard and index test. All studies showed that the index test was conducted exactly as the review question and that the reference standard defined the target condition matching the review question (Table 2) [24].

All studies showed that the surgical procedures were done with the knowledge of the radiological findings; this explains the high risk of bias as in regard to the reference standard. This bias was inevitable because it is difficult to blind the surgeons about MRI findings (Table 2).

There was some heterogeneity between studies, so we tried to decrease it by pooling results of studies with similar types of MRI, strength of magnetic field machines and lesion types (Table 4). There was some variability between studies in assessors, imaging planes, sequences, slice thicknesses and resolution. These factors were too complex to be analyzed as part of this meta-analysis and this should be considered when interpreting the pooled results.

We believe that MRI is highly depended on the radiologists who read its images, this factor could explain also the heterogeneity of results even between similar studies. The difference between the accuracy of radiologists still needing further studies and there was insufficient data to perform meta-analysis on this point.

Only one study used 1 T magnet [15], this low field strength may be not appropriate hip because it is large and deep joint. Three studies used 3 T magnet [14, 21, 31] but it was not possible to get pooled data from them because each study used different MRI type. So all the 12 studies included in the meta-analysis used 1.5 T magnetic field strength.

Tian et al. [14] concluded that dMRA at 3.0 T was more accurate method for diagnosing acetabular labral tears, with a significant greater sensitivity and NPV compared with cMRI examination, but in their study only 30% of the study population did dMRA while 100% did MRI. This was the only study using 3.0 T magnet for dMRA and cMRI and that is why it was not possible to do a meta-analysis on the difference between MRI fields.

Gonzalez et al. [31] found 87% sensitivity and 77% specificity for the diagnosis of labral lesions using dMRA. For the chondral lesions they found lower values in both locations, acetabular and femoral.

These studies showed good results using high field strength magnet and this were explained by increasing the signal-to-noise ratio thus helping in detailed assessment of intraarticular structures such as labrum and cartilage [41, 42]. More investigation is therefore required for the 3 T imaging to precisely detect its accuracy.

There are a number of disadvantages for dMRA because the injection of gadolinium directly into the joint is an invasive procedure and carries small risk of joint infection [43]. Also the use of contrast material increases both the cost and the time of dMRA examination over cMRI.

### Labral lesions

The number of studies included in the meta-analysis for dMRA were 8 and for cMRI were 3 studies (Figs. 2, 3, 6 and 7). The results showed that the diagnostic test accuracy was superior for dMRA when compared with cMRI for detection of labral lesions (Table 4).

Similar results were achieved by a meta-analysis done by Smith et al. [19] which showed that the pooled sensitivity and specificity for cMRI for diagnosing acetabular labral tears were 66% and 79% and for dMRA were 87% and 64% however, the data in that meta-analysis lack analysis of iMRA studies and include all causes of labral pathologies with no specificity to FAI as in this review.

Sutter et al. [12] showed that dMRA arthrography showed an advantage over cMRI in the detection of labral tears for one reader, whereas both methods were equivalent for the other reader. These results shows that the MRI procedure is operator dependent and this was confirmed in another study, McGuire et al. [13] showed that musculoskeletal (MSK) specialists had more accuracy than general radiologists in detecting labral lesions and also showed a higher accuracy of dMRA in detecting labral lesions when analyzing both groups of radiologists in comparison with cMRI.

The previous 2 papers [12, 13] compared the accuracy of dMRA with cMRI and they concluded that dMRA had higher accuracy than cMRI in detecting labral lesions which matched the results of this meta-analysis.

Keeney et al. [44] showed that a negative result of dMRA study does not exclude important intraarticular pathology that can be identified and managed. With respect to labral lesions they showed a sensitivity of 71%, specificity of 44%. They stated that the assessment of specificity of dMRA in the evaluation of labral lesions was limited because of the small number of patients without acetabular labral tears in their study.

Reurink et al. [34] showed that the overall sensitivity and specificity for detecting labral lesions were 86% and 75%. They concluded that dMRA has a poor Negative predictive value and cannot be used to rule out a labral tear when there is a high clinical suspicion of such a tear which matches the results of this meta-analysis.

### Chondral lesions

The number of studies included in the meta-analysis for dMRA were 8 studies and for cMRI studies were 3 studies (figs. 4,5,8 and 9). The results showed that the diagnostic test accuracy was superior for dMRA when compared with cMRI for detection of chondral lesions (Table 4).

Smith et al. [18] achieved different results in their meta-analysis, they concluded that the accuracy for the diagnosis of hip joint chondral lesions is higher for cMRI compared to dMRA but the data in that meta-analysis lack analysis of iMRA studies and include all causes of chondral pathologies with no specificity to FAI. There was some heterogeneity by pooling results from studies using different MR strength fields. They found that the pooled sensitivity and specificity for cMRI for diagnosing chondral lesions were 59% and 94% and for dMRA were 62% and 86%.

Sutter et al. [12] showed that dMRA was superior to cMRI for detecting acetabular cartilage defects but for femoral cartilage lesions, both modalities yielded comparable results. They indicated that both dMRA and cMRI allow identification of the patients with extensive cartilage damage at the acetabular rim. For patients with non-extensive cartilage damage at the acetabular rim dMRA showed increased accuracy compared with cMRI.

McGuire et al. [13] showed that MSK radiologists performed better than community radiologists in terms of overall accuracy. Accuracy rates for MSK radiologists were 79 and 59 for acetabular chondral lesions and femoral chondral lesions, respectively, whereas accuracy rates for community radiologists were 28 and 52%. Accuracy was significantly increased for both groups of radiologists when dMRA were reviewed rather than cMRI and concluded that dMRA has been shown to be more sensitive and specific for diagnosing hip joint pathology.

The previous 2 studies compared the accuracy of dMRA with cMRI and they concluded that dMRA had higher accuracy than cMRI in detecting of chondral lesions and this matched the results of this meta-analysis.

Keeney et al. [44] showed that with respect to chondral lesions, dMRA had a sensitivity of 47%, specificity of 89%, and an accuracy of 67%. Also Aprato et al. [33] showed that the role of dMRA in evaluating chondral lesions is limited. The sensitivity for femoral chondral lesions was 46%, specificity was 81% and for acetabular cartilage injuries, the sensitivity was 69%, Specificity was 88%. These data show that negative dMRA study should not rule out the presence of chondral lesion if it was clinically suspected.

Four studies [12, 13, 33, 38] assessed the accuracy of dMRA in detection of acetabular chondral lesions, the pooled sensitivity and specificity for these studies were 0.86 and 0.68 respectively, and AUC was 0.86. Four studies [12, 13, 33, 38] assessed the accuracy of dMRA in detection of femoral head chondral lesions, the pooled sensitivity and specificity for these studies were 0.69 and 0.75 respectively, and AUC was 0.75. Two studies [36, 37] assessed the accuracy of dMRA in detection of combined chondral lesions, the pooled sensitivity and specificity for these studies were 0.51 and 0.88 respectively.

Two studies [12, 13] assessed the accuracy of cMRI in detection of acetabular chondral lesions, the pooled sensitivity and specificity for these studies were 0.84 and 0.88 respectively. Two studies [12, 13] assessed the accuracy of cMRI in detection of femoral head chondral lesions, the pooled sensitivity and specificity for these studies were 0.73 and 0.85 respectively.

This analysis of different chondral lesions showed close results except for Femoral head chondral lesions which was the lowest value and this could be explained by the tight congruence hip joint and difficult recognition of femoral head cartilage.

### iMRA

We performed an analysis of iMRA but the studies were few in number, only 2 studies for chondral lesions provided suitable data for meta-analysis [21, 35]. The pooled analysis indicated Sensitivity of 0.722 (95% CI: 0.465 – 0.903), specificity of 0.917 (95% CI: 0.615 – 0.998). As in regard to chondral lesions, iMRA showed acceptable results like dMRA but further studies are still needed concerning this technique to get more reliable data (Table 4).

**Table 4:** Summary of pooled results with 95% CI for accuracy of dMRA, cMRI and iMRA in detecting chondral and labral hip lesions

Analysis	N	Sensitivity	Specificity
*dMRA*			
Chondral lesions	8	0.75 (95% CI: 0.69 – 0.8)	0.866 (95% CI: 0.789 – 0.923)
Labral lesions	8	0.91 (95% CI: 0.88 – 0.94)	0.58(95% CI: 0.48 – 0.68)
*cMRI*			
Chondral lesions	3	0.76 (95% CI: 0.65 – 0.85)	0.72 (95% CI: 0.57 – 0.84)
Labral lesions	3	0.864 (95% CI: 0.757 – 0.936)	0.833 (95% CI: 0.359 – 0.996)
*iMRA*			
Chondral lesions	2	0.722 (95% CI: 0.465 – 0.903)	0.917 (95% CI: 0.615 – 0.998)
Labral lesions	2	N/C	N/C

*N* number of data sets, *N/C* not calculable, *LR* Likelihood ratio

It was not possible to analyze data concerning labral tears as it was incalculable. The two studies that present data about labral lesions using iMRA reported sensitivity and specificity of (0.89–0.99) in Petchprapa et al. [21] and (1.00-1.00) in Zlatkin et al. [35], these results are considered very high and indicate that further studies concerning iMRA are still needed.

### dGEMRIC

The accuracy of dGEMRIC in diagnosing hip joint chondral lesions was discussed in three studies [27, 28, 40]. However, there were insufficient data to do meta-analysis since only one study [40] showed the needed data for analysis, the results indicated a sensitivity of (0.75,0.52,0.71) and specificity of (0.33,0.67,0.36) respectively.

Lattanzi et al. [27] suggest that Standardized dGEMRIC at 3.0 T is repeatable and could considerably improve preoperative assessment of hip articular cartilage, a critical component in the decision making process for the surgeon and patient considering hip arthroscopy for FAI. Results showed that the sensitivity, specificity and accuracy for Observer 1 and Observer 2, were 83%, 60% and 75%, and 69%, 70% and 69%, respectively. Overall performance was 52%, 67% and 58%.

Lattanzi et al. [28] reported sensitivity, specificity and accuracy of 47%, 58% and 55% for dGEMRIC and 47%, 79% and 70% for morphologic evaluation, respectively. They suggested that standardized dGEMRIC may be able to detect chondral lesions with high sensitivity and accuracy in FAI patients.

The results presented here suggest that adding standardized dGEMRIC to morphologic chondral evaluation could help surgical decisions in FAI, this new quantitative MRI technique have great potential to diagnose early chondral and labral lesions by detecting changes in chondral thickness and volume, as well as proteoglycan, collagen and water content. Also further studies concerning dGEMRIC are still needed.

## Conclusions

The results of this review meta-analysis show that MRI, dMRA and iMRA are useful tools in diagnosing labral and chondral lesions of the hip joint with FAI.

The diagnostic test accuracy was superior for dMRA when compared with cMRI for detection of labral and chondral lesions.

Detection of chondral lesions had low accuracy as compared with labral lesions in both cMRI and dMRA. Promising results are obtained concerning iMRA but further studies still needed to fully assess its diagnostic accuracy.

The new dGEMERIC protocol and 3.0 T field strength magnets could be interesting for further study, also the difference between the accuracy of radiologists still needs further analysis.

## Additional files

Additional file 1: Meta-disc file for chondral lesions in dMRA. Statistical file from Meta-disc program showing true positives, true negatives, false positives and false negatives results of direct magnetic resonance arthrography in detecting chondral lesions. (DSC 80 kb)
Additional file 2: Meta-disc file for labral lesions in dMRA. Statistical file from Meta-disc program showing true positives, true negatives, false positives and false negatives results of direct magnetic resonance arthrography in detecting labral lesions. (DSC 80 kb)
Additional file 3: Meta-disc file for chondral lesions in cMRI. Statistical file from Meta-disc program showing true positives, true negatives, false positives and false negatives results of conventional magnetic resonance imaging in detecting chondral lesions. (DSC 80 kb)
Additional file 4: Meta-disc file for labral lesions in cMRI. Statistical file from Meta-disc program showing true positives, true negatives, false positives and false negatives results of conventional magnetic resonance imaging in detecting labral lesions. (DSC 80 kb)

## Abbreviations

AUC: Area under receiver operating characteristic curve
CI: Confidence intervals
cMRI: Conventional magnetic resonance imaging
dGEMRIC: Delayed gadolinium enhanced magnetic resonance imaging of cartilage
dMRA: Direct magnetic resonance arthrography
FAI: Femoroacetabular impingement
FN: False-negatives
FP: False-positives
iMRA: Indirect magnetic resonance arthrography in the detection of chondral
MeSH: Medical subject headings
MR: Magnetic resonance
MRI: Magnetic resonance imaging
PRISMA: Preferred reporting items for systematic reviews and meta-analyses
QUADAS: Quality assessment of diagnostic accuracy studies
ROC: Receiver operating characteristic
T: Tesla
TN: True-negatives
TP: True-positives
